# Deceased donor interposition graft repair of superior mesenteric artery injury during cytoreductive nephrectomy for metastatic renal cell carcinoma

**DOI:** 10.1093/jscr/rjag876

**Published:** 2026-10-10

**Authors:** Nima Iranpour, Victor Yu, Fatima Gutierrez, Emily Smartt, Alison Zhang, John Boulas, Jerome Laurence

**Affiliations:** Department of Urology, Royal Prince Alfred Hospital, 50 Missenden Road, Camperdown, NSW 2050, Australia; Department of Urology, Royal Prince Alfred Hospital, 50 Missenden Road, Camperdown, NSW 2050, Australia; Department of Medical Oncology, Chris O’Brien Lifehouse, 119–143 Missenden Road, Camperdown, NSW 2050, Australia; Department of General Surgery, Royal Prince Alfred Hospital, 50 Missenden Road, Camperdown, NSW 2050, Australia; Department of Medical Oncology, Chris O’Brien Lifehouse, 119–143 Missenden Road, Camperdown, NSW 2050, Australia; Department of Urology, Royal Prince Alfred Hospital, 50 Missenden Road, Camperdown, NSW 2050, Australia; Department of General Surgery, Royal Prince Alfred Hospital, 50 Missenden Road, Camperdown, NSW 2050, Australia; Department of Transplant Surgery, Royal Prince Alfred Hospital, 50 Missenden Road, Camperdown, NSW 2050, Australia

**Keywords:** carcinoma, renal cell, organ transplantation, transplant rejection, immune checkpoint inhibitors, nephrectomy

## Abstract

Iatrogenic injury to the superior mesenteric artery (SMA) represents a rare but serious complication of nephrectomy, associated with significant morbidity and mortality. Repair can be technically challenging. We describe the case of a 71-year-old patient who had an SMA injury during an open nephrectomy for a large carcinoma. Her SMA was reconstructed successfully using a deceased-donor iliac artery interposition graft. Due to her high-risk pathology and progressive disease early in the post-operative period, she was commenced on lenvatinib and pembrolizumab. Despite the vascular allograft, immunosuppression was not used due to concerns about an interaction with immunotherapy. To date, she has had an excellent treatment response with a significant reduction in metastatic burden without the development of rejection of the vascular allograft, despite no immunosuppression.

## Introduction

Iatrogenic ligation of the superior mesenteric artery (SMA) is a rare but devastating complication during left-sided radical nephrectomy with a mortality rate up to 75% [1]. The highest risk of such injury often occurs in the setting of nephrectomy for advanced renal cell carcinoma (RCC), particularly in the context of bulky para-aortic lymphadenopathy [2] where adjuvant immunotherapy has demonstrated the best survival outcome long-term [3]. We report the first case of the use of deceased donor interposition graft to reconstruct the SMA and the subsequent use of adjuvant immunotherapy without requiring immunosuppression or eliciting a rejection response.

## Case report

A 71-year-old female was consented to an open left radical nephrectomy and retroperitoneal lymphadenectomy for a 22 cm left upper pole heterogenous, mixed-solid cystic mass demonstrated on magnetic resonance imaging (MRI) and computed tomography (CT). Salient imaging findings also included a single left renal artery and vein without tumour involvement and a 39 mm pararenal nodal mass (Fig. 1a). Notably, CT angiography also demonstrated a pre-existing coeliac occlusion with collateralization of foregut structures via SMA. She had no prior abdominal or renal surgery and no other significant comorbidities.

**Figure 1:**
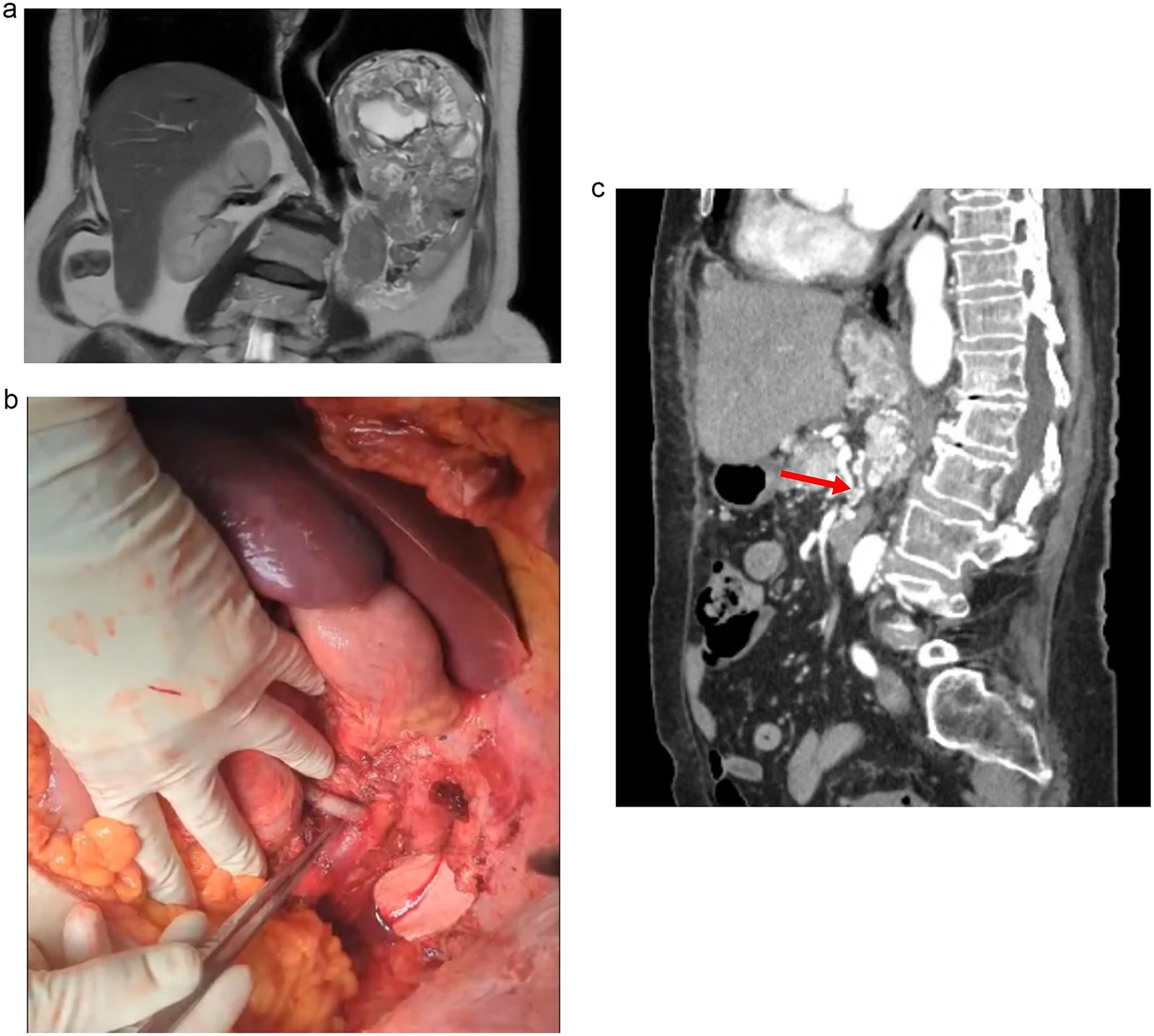
(a) Pre-operative coronal MRI demonstrating a large upper pole tumour with close relation to the abdominal aorta, (b) intra-operative photograph of donor interposition, and (c) post-operative sagittal CT demonstrating patent SMA.

She proceeded to an open left transperitoneal nephrectomy, where mobilization of the kidney was complicated by her bulky hilar lymphadenopathy and distorted aorta. During dissection and control of the hilum, a vessel initially thought to be the main renal artery was ligated with a stapler but was later intraoperatively identified as the SMA. Attempted primary repair of the transected SMA was performed; however, it was unsuccessful due to friability of tissue. With vascular and transplant surgeon involvement, a decision was made to reconstruct the SMA using a deceased donor iliac vessel (blood group compatible, negative viral serology). End-to-end anastomosis was performed proximally and distally, and following unclamping, macroscopically good bowel perfusion was demonstrated (Fig. 1b). The coeliac trunk and reconstructed SMA were interrogated with intraoperative ultrasound which showed preserved vascular flow.

The patient was discharged home following an uncomplicated course 3 weeks post-operatively. A re-staging CT with angiography performed at 1 month confirmed SMA patency (Fig. 1c); however, it demonstrated significant intraperitoneal and pleural metastases. Histopathology revealed clear-cell RCC with lymphatic and venous invasion and adrenal extension with 2/11 positive lymph nodes. Based on International Metastatic RCC Database Consortium risk score [4], she was deemed poor risk at 6/6 with a median predicted survival of 7.8 months.

Upon multidisciplinary discussion with the patient and family, and given her poor prognosis, a decision was made to proceed with systemic therapy, acknowledging the risks of thrombosis and the potential for allograft rejection. One month post-operatively she was commenced on lenvatinib (20 mg daily) and pembrolizumab (200 mg every 21 days). The patient had a good treatment response at 3 and 6 months, with a progressive size reduction in her metastatic burden observed on CT. She remains well at 8 months post-operatively, with graft patency maintained on aspirin and subcutaneous enoxaparin, and free from immunosuppression or evidence of allograft rejection since commencement of immunotherapy.

Two years post-resection, the patient is still alive. Her CT is showing no thrombosis or narrowing of the graft and oncologically she has stable disease on the same combination immunotherapy without immunosuppression.

## Discussion

This case presents a number of important clinical considerations and challenges not uncommonly faced in the context of metastatic RCC. Although rare, awareness of the potential for SMA injury should always be considered in left-sided nephrectomy, particularly in cases of large renal tumours with local lymphadenopathy and distorted anatomy. Described methods of repair include direct anastomosis, aortic reimplantation, autologous or prosthetic vein grafts, and splenic arterial bypasses [2, 5]—with varied reported success rates. This case is the first known use of a deceased donor vessel as an interposition graft to repair an iatrogenic SMA ligation during nephrectomy.

Importantly, this case highlights the safety of using deceased donor vascular grafts in an oncological context, including advanced RCC. Despite survival benefits [6], use of combination tyrosine kinase inhibitors (TKI) and immune checkpoint inhibitors (ICI) can be problematic in the context of allografts, with a potentially increased risk for rejection. Literature describing their use in transplants is limited; however, the use of pembrolizumab has been associated with the highest rates of graft rejection amongst ICIs, up to 39%, though in the context of solid organ transplants receiving immunosuppression [7]. No practice guidelines have yet emerged to guide ICI use in isolated vascular allografts. Some studies describe use of isolated vascular allografts without immunosuppression, often in conjunction with specific anticoagulation protocols [8] while others have adopted the use of low-dose immunosuppression, administered for prolonged periods (1-year or indefinitely) [9]. In our case, no induction or maintenance immunosuppression was utilized, which appears safe, given the lack of evidence of rejection to date. TKI scan induces vascular thrombosis [10], but this has not been a complication of the allograft, even with anticoagulation and antiplatelet therapy. ICIs are becoming the mainstay systemic therapy in RCC, and indications continue to expand rapidly [11]; however, more evidence is required to guide their use in transplant, particularly surveillance and indications for immunosuppression.

## Conclusion

Overall, our case highlights the use of a deceased donor allograft as an option in repair of an iatrogenic SMA injury. Importantly, we also demonstrate the safety of immunotherapy in the context of a vascular allograft, without compromising oncological outcomes.

## References

[1] Asensio JA, Britt LD, Borzotta A et al. Multiinstitutional experience with the management of superior mesenteric artery injuries. J Am Coll Surg 2001;193:354–65. discussion 65–6. 10.1016/s1072-7515(01)01044-4 11584962

[2] Catarci M, Montemurro LA, Benedetti M et al. Extrarenal visceral arteries injuries during left radical nephrectomy: a 50-year continuing problem. J Clin Med 2024;13:6125. 10.3390/jcm13206125 PMC11508894 39458075

[3] Choueiri TK, Tomczak P, Park SH et al. Overall survival with adjuvant pembrolizumab in renal-cell carcinoma. N Engl J Med 2024;390:1359–71. 10.1056/NEJMoa2312695 38631003

[4] Heng DY, Xie W, Regan MM et al. External validation and comparison with other models of the international metastatic renal-cell carcinoma database consortium prognostic model: a population-based study. Lancet Oncol 2013;14:141–8. 10.1016/S1470-2045(12)70559-4 PMC4144042 23312463

[5] Sayegh AS, Medina LG, La Riva A et al. Superior mesenteric artery injury during robotic radical nephrectomy: scenarios and management strategies. J Clin Med 2023;12:427. 10.3390/jcm12020427 PMC9865815 36675356

[6] Bex A, Ghanem YA, Albiges L et al. European Association of Urology guidelines on renal cell carcinoma: the 2025 update. Eur Urol 2025;87:683–96. 10.1016/j.eururo.2025.02.020 40118739

[7] Kumar V, Shinagare AB, Rennke HG et al. The safety and efficacy of checkpoint inhibitors in transplant recipients: a case series and systematic review of literature. Oncologist 2020;25:505–14. 10.1634/theoncologist.2019-0659 PMC7288631 32043699

[8] Bossi M, Tozzi M, Franchin M et al. Cryopreserved human allografts for the reconstruction of aortic and peripheral prosthetic graft infection. Ann Vasc Dis 2017;10:391–7. 10.3400/avd.oa.17-00068 PMC5835436 29515701

[9] Pluchova M, Chlupac J, Janousek L et al. Fresh arterial allograft as a replacement for an infected common femoral prosthetic graft and recurrent false aneurysm. Rozhl v Chir 2023;102:75–9. 10.33699/PIS.2023.102.2.75-79 37185029

[10] Qi W-X, Shen Z, Tang L-N et al. Risk of arterial thromboembolic events with vascular endothelial growth factor receptor tyrosine kinase inhibitors: an up-to-date meta-analysis. Crit Rev Oncol Hematol 2014;92:71–82. 10.1016/j.critrevonc.2014.04.004 24878433

[11] Twomey JD, Zhang B. Cancer immunotherapy update: FDA-approved checkpoint inhibitors and companion diagnostics. AAPS J 2021;23:39. 10.1208/s12248-021-00574-0 PMC7937597 33677681

